# Circadian preference in children and adolescents with migraine - a controlled study

**DOI:** 10.1186/1129-2377-14-S1-P25

**Published:** 2013-02-21

**Authors:** G Gelbmann, C Wöber-Bingöl

**Affiliations:** 1 Department of Child and Adolescent Psychiatry, Medical University of Vienna, Austria

## Introduction

Research on chronobiological aspects of neurological disorders has gained influence in recent time. Besides epilepsy, dementia and movement disorders [1] migraine was identified to be influenced by the circadian clock [2].

## Purpose/background/objectives

The aim of the present study was to investigate the circadian preference of children and adolescents with migraine.

## Methods

We compared circadian preference of patients with migraine according to the criteria of ICHD-2 with that of headache-free controls matched for age and sex. For differentiating morning-, intermediate and evening-types we applied the Morningness-Eveningness Questionnaire.

## Results

We included 67 children (age 6-11) and 78 adolescents (aged 12-18) with migraine as well as a total of 244 headache-free controls. In children, we found significant differences between patients and controls (χ²=37.075, df=2, p<0.001). Morningness as well as eveningness tendencies were more common in subjects with migraine than in controls. In contrast, the circadian preference of adolescents with and without migraine did not differ from each other(χ²=0.833, p=0.659).

## Conclusion

Children with migraine tended towards extremer circadian orientation, but this was not the case in adolescents. As eveningness is connected with sleeping and emotional problems and morningness seems to have a protective function concerning the development of sleeping and emotional problems, these findings may be seen as starting point for possible new therapeutical interventions such as specific psychoeducational strategies, light- and chronotherapy in children with migraine.
